# Clinical and demographic parameters predict the progression from mild cognitive impairment to dementia in elderly patients

**DOI:** 10.1007/s40520-020-01697-8

**Published:** 2020-09-12

**Authors:** Giovanni Zuliani, Michele Polastri, Tommaso Romagnoli, Lisa Marabini, Davide Seripa, Carlo Cervellati, Amedeo Zurlo, Angelina Passaro, Gloria Brombo

**Affiliations:** 1 Department of Morphology, Surgery, and Medical Sciences, University of Ferrara, Azienda Ospedaliero-Universitaria S. Anna, 44100 Ferrara, Italy; 2 Department of Medical Sciences, University of Ferrara, Ferrara, Italy; 3 Research Laboratory, Complex Structure of Geriatrics, Department of Medical Sciences, Fondazione IRCCS Casa Sollievo Della Sofferenza, San Giovanni Rotondo, Italy; 4 Department of Biomedical and Specialist Surgical Sciences, University of Ferrara, Ferrara, Italy

**Keywords:** Aging, Cox regression, Dementia, Follow-up, Mild cognitive impairment

## Abstract

**Objectives:**

To evaluate the possibility of predicting the risk of progression from mild cognitive impairment (MCI) to dementia using a combination of clinical/demographic parameters.

**Methods:**

A total of 462 MCI elderly patients (follow-up: 33 months). Variable measured included cognitive functions, age, gender, MCI type, education, comorbidities, clinical chemistry, and functional status.

**Results:**

Amnestic type (aMCI) represented 63% of the sample, non-amnestic (naMCI) 37%; 190 subjects progressed to dementia, 49% among aMCI, and 28% among naMCI. At Cox multivariate regression analysis, only MMSE (one point increase HR 0.84; 95% CI 0.79–0.90), aMCI (HR 2.35; 95% CI 1.39–3.98), and age (1 year increase HR 1.05; 95% CI 1.01–1.10) were independently associated with progression to dementia. A score was created based on these dichotomized variables (score 0–3): age (≥ or < 78 years), MMSE score (≥ or < 25/30) and aMCI type. The conversion rate progressed from 6% in subjects with score 0 (negative predictive value: 0.94), to 31% in individuals with score 1, to 53% in subjects with score 2, to 72% in individuals with score 3 (positive predictive value: 0.72). ROC curve analysis showed an area under the curve of 0.72 (95% CI 0.66–0.75, *p* 0.0001).

**Conclusions:**

We have described a simple score, based on previously recognized predictors such as age, MMSE, and MCI type, which may be useful for an initial stratification of the risk of progression to dementia in patients affected by MCI. The score might help the clinicians to evaluate the need for more expansive/invasive examinations and for a closer follow-up in MCI patients.

**Electronic supplementary material:**

The online version of this article (10.1007/s40520-020-01697-8) contains supplementary material, which is available to authorized users.

## Introduction

Mild cognitive impairment (MCI) is an intermediate condition in the trajectory from normal cognition to dementia; compared to normal individuals, subjects with MCI usually have a higher rate of progression to dementia over a relatively short period [1]. The operational criteria for MCI diagnosis are the presence of a cognitive complain/impairment, an objective evidence of impairment in cognitive domains (e.g., memory, executive function/attention, language or visuospatial skills), essentially normal function in activities of daily living, and absence of dementia [1]. The reported prevalence of MCI over 60 years of age is approximately 7–25% [2]; it increases with age and lower level of education, and seem to be more prevalent in men [2].

However, the prevalence of the preclinical phase of dementia may vary greatly, according to the diagnostic criteria and assessment procedures. In Italy, it has been estimated that up to 45% of population aged 65–84 years had some kind of cognitive deficits without dementia [3].

Basically, a presentation with memory impairment is typical of the amnestic form of MCI (aMCI), whereas non-amnestic MCI (naMCI) is characterized by impairment in non-memory cognitive domains [1]. Currently, the majority of researchers employ four subtypes of MCI, depending on the number of affected domains: single-domain aMCI, multidomain aMCI, single-domain naMCI, and multidomain naMCI [4].

The issue of the progression to dementia is crucial, since the diagnosis of MCI implicates a prognosis that is “less favorable” compared to persons with normal cognition. Most of the studies report a rate of progression from 20 to 40%, with an annual rate of 5–17% [1, 2]. However, while most patients who will develop dementia will exhibit symptoms compatible with MCI in the earlier stages of the disease [5], the reverse may not be true, since many individuals with MCI diagnosis may never progress to dementia [6]. Moreover, a small percentage of MCI individuals may revert to normal cognition, and even among these subjects, the risk of subsequent MCI or dementia is higher compared to healthy controls [7]. aMCI is hypothesized to progress mostly to Alzheimer’s disease (AD), while naMCI usually progress to non-AD forms of dementia; both naMCI and aMCI have been associated with progression to vascular dementia (VD) [1]. Several risk factors have been associated with the possible progression from MCI to overt dementia in Literature including: older age, lower levels of formal education, memory loss or aMCI type, multiple domain MCI type, previous stroke, cerebrovascular disease, cardiovascular risk factors (e.g., hypertension, diabetes), presence of depression, poor health status, the degree of cognitive impairment, and perhaps female gender [1].

In the present study we evaluated, in a large sample of older individuals affected by MCI, the possibility of predicting the progression to dementia using a combination of easily accessible and inexpensive clinical/demographic parameters.

## Patients and methods

### Subjects

The initial sample included 620 elderly (≥ 65 years) outpatients evaluated in the period 2006–2018 at the Memory Clinic of the Department of Internal Medicine, S. Anna University Hospital, Ferrara (Italy) or of the Casa Sollievo della Sofferenza, San Giovanni Rotondo (Italy) in which the diagnosis of MCI was made. After the initial examination and diagnosis, 158 subjects were lost during follow-up (we had no evidence from computerized health care system of a possible diagnosis of dementia), while 462 underwent a regular follow-up (median: 33 months; range 10–155). Of these, 190 converted to dementia, with a final rate of progression of about 30% (190/620).

Subjects lost at follow-up did not differ from individuals included into the study as regards age, gender, education, MCI type, or prevalence of diabetes, hypertension, coronary heart disease (CHD), or stroke (data not shown).

MCI was defined as the presence of a documented deficit in memory or in another cognitive domain, without (single domain) or with (multiple domain) impairment in other cognitive domains, in an individual who didn’t meet the clinical criteria for dementia [4].

The Mini-Mental State Examination (MMSE) [8] ranged from 22/30 to 27/30.

The clinical diagnosis of dementia during follow-up was made by the DSM-5 criteria [9].

LOAD diagnosis was based on the National Institute on Aging-Alzheimer’s Association workgroups criteria [10], while the diagnosis of VD was made following the NINDS-AIREN criteria [11].

There were no evidence of acute illnesses at the time of clinical observation and blood sampling, as previously described [12, 13]. No subject was taking NSAIDS, antibiotics, or steroids at the time of recruitment.

A cardiovascular risk score was calculated as follows: 1 point for active smoking, diabetes, hypertension and male gender; 2 points for previous diagnosis of coronary heart diseases or stroke (total score 0–8).

General and neuropsychological examination including basic activities of daily living (BADLs) [14], instrumental activities of daily living (IADLs) [15], and 15 items geriatric depression scale (GDS) [16] was carried as previously described [17]. Personal data and medical history (e.g., hypertension, coronary heart disease—CHD, diabetes, chronic obstructive pulmonary disease—COPD) were collected by trained personnel as previously described [18]. Clinical chemistry analyses were routinely performed to exclude causes of secondary cognitive impairment. These analyses included serum B-12 vitamin and folate, liver, kidney and thyroid function tests, blood cell count, and arterial oxygen saturation.

All subjects underwent brain MRI or brain CT using a 64 volumetric scanner.

The study was approved by the Local Ethic Committee of “Casa Sollievo della Sofferenza”, San Giovanni Rotondo (protocol n. 3877/DS) and Local Ethic Committee of “Azienda Arcispedale S. Anna”, Ferrara (protocol n. 170579). Patients were informed about the project and research protocol, and a written consent was obtained. The research did not modify the routine clinical/diagnostic protocols implemented for the diagnosis of MCI nor conditioned any decision about the treatments of the enrolled individuals.

### Statistical analysis

Continuous variables were expressed as mean (standard deviation—SD) or median (interquartile range—IQR) when necessary. Means were compared by ANOVA with Bonferroni post hoc test for multiple comparison; medians were compared by Mann–Whitney test. Correlations between continuous variables were tested by Pearson’s correlation. Proportions were compared by the *χ*^2^ test. Hazard ratios (HR) were estimated by Cox proportional hazard regression analysis (univariate and multivariate). The assumption of proportionality of all variables introduced in the models was assessed through the analysis of Schoenfeld residuals. Analyses were performed by SPSS for Windows statistical package, version 13.0.

## Results

Most of the sample was composed by aMCI patients (n.292–63%), while the remaining subjects were affected by naMCI (n.170–37%). The most frequent diagnosis was multidomain aMCI (54%), followed by multidomain naMCI (25%), single-domain naMCI (12%), and single-domain aMCI (9%). On the whole, 190 MCI progressed to dementia during the follow-up, about one in two among aMCI (49%), and one in three among naMCI (28%); in particular, the rate of progression was 29% for multiple naMCI, 34% for single naMCI, 48% for single aMCI, and 52% for multiple aMCI (Supplementary Fig. 1). Among the patients progressed to dementia, 34% developed LOAD, 35% “mixed” dementia (dementia with clinical–instrumental characteristics of both LOAD and VD), 29% VD, and 2% other forms of dementia.

In Table 1 are reported the principal characteristics of the sample according to clinical evolution. Compared to stable MCI, MCI converted to dementia were characterized by older age, higher prevalence of amnestic type, lower MMSE score, less depressive symptoms, and lower sub-cortical multiple lacunes. A non-significant trend toward an increase in the prevalence of diabetes, leukoaraiosis and brain atrophy was also observed.

**Table 1:** Principal characteristics of the sample according to follow-up evolution

	MCI–MCI (*n* 272)	MCI–dementia (*n* 190)	*p*
Demographics			
Age (years)	76.6 ± 6.3	78.3 ± 4.7	0.002
Female gender (%)	54	59	0.33
Education (years)	6.1 ± 3.7	6.0 ± 3.4	0.75
Active smoking (%)	7.8	8.0	0.96
Cognitive and functional status			
MMSE (/30)	25.3 ± 2.7	24.0 ± 2.8	0.001
GDS (/15)	5 (3–7)	3 (2–6)	0.007
BADLs (/6)	5.4 ± 1.0	5.1 ± 1.2	0.80
IADLs (/8)	6.4 ± 2.1	5.9 ± 2.5	0.18
Amnestic type MCI (within group) (%)	55	74.5	0.001
Clinical chemistry parameters			
Haemoglobin (g/dl)	13.1 ± 1.4	13.1 ± 1.4	0.98
Creatinine (mg/dl)	0.94 ± 0.27	0.95 ± 0.29	0.86
Uric acid (mg/dl)	4.9 ± 1.6	5.1 ± 1.2	0.64
Albumin (g/dl)	4.0 ± 0.33	4.0 ± 0.35	0.59
Total cholesterol (mg/dl)	205 ± 40	207 ± 41	0.68
Tryglicerides (mg/dl)	107 ± 38	119 ± 67	0.0
HDL-C (mg/dl)	60 ± 16	64 ± 38	0.23
Hs-CRP (mg/dl)	0.18 (0.12–0.25)	0.16 (0.10–0.27)	0.46
Glycemia (mg/dl)	98 ± 21	103 ± 41	0.25
HbA1c (%)	5.9 ± 0.8	6.1 ± 1.3	0.15
Cardiovascular Score	1.68 ± 1.1	1.64 ± 1.1	0.75
Homocysteine (μmol/l)	15 (12–18)	16 (14–20)	0.10
Vitamin B12 (pg/ml)	302 (247–387)	309 (263–283)	0.92
Folic acid (ng/ml)	6 (5–7)	5.9 (5.1–7)	0.78
Comorbidities			
Hypertension (%)	56	58	0.97
Coronary heart disease (%)	14.2	13.5	0.86
Diabetes (%)	14.8	20.1	0.15
Stroke (%)	4.1	5.3	0.64
COPD (%)	6.1	6.1	0.98
MRI parameters			
Cortical lesions (%)	13.6	12.8	0.67
Single lacunar lesion (%)	7	8	0.72
Multiple lacunar lesions (%)	42	31	0.04
Leukoaraiosis (%)	33	42	0.14
Atrophy (%)	49	57	0.21

Continuous variables are expressed as mean ± SEM or median (interquartile range). Categorical variables are expressed as percentage within group

*MMSE* mini mental state examination, *GDS* global deterioration scale, *IADL* instrumental activities of daily living, *BADL* basic activity of daily living, *HDL-C* high density lipoprotein cholesterol, *Hs-CRP* high-sensitivity C-reactive protein, *HbA1c* hemoglobin A1c

In Table 2 are described the principal characteristics of the sample according to the clinical type of MCI. Compared with naMCI patients, aMCI were older, had less depressive symptoms, and lower hsCRP levels. The calculated cardiovascular score was generally low in the whole sample and did not differ between stable MCI patients or patients progressing to dementia, nor between aMCI and naMCI.

**Table 2:** Principal characteristics of the sample according to MCI type

	Amnestic MCI (*n* 292)	Non-amnestic MCI (*n* 170)	*p*
Demographics			
Age (years)	77.7 ± 5.6	76.2 ± 5.8	0.01
Female gender (%)	57	61	0.24
Education (years)	6.0 ± 3.4	6.1 ± 3.6	0.77
Active smoking (%)	8.5	8.1	0.96
Cognitive and functional status			
MMSE (/30)	24.6 ± 2.7	24.2 ± 3.3	0.18
GDS (/15)	4 (3–6)	5 (3–6)	0.05
BADLs (/6)	5.4 ± 1.0	5.2 ± 1.3	0.85
IADLs (/8)	5.9 ± 2.4	5.6 ± 2.7	0.40
Clinical chemistry parameters			
Haemoglobin (g/dl)	13.1 ± 1.4	13.1 ± 1.4	0.98
Creatinine (mg/dl)	1.01 ± 0.27	0.94 ± 0.29	0.74
Uric acid (mg/dl)	5.0 ± 1.8	5.3 ± 1.4	0.41
Albumin (g/dl)	4.0 ± 0.37	4.0 ± 0.31	0.61
Total cholesterol (mg/dl)	207 ± 45	207 ± 40	0.97
Tryglicerides (mg/dl)	117 ± 54	116 ± 57	0.82
HDL-C (mg/dl)	61 ± 15	58 ± 29	0.28
Hs-CRP (mg/dl)	0.15 (0.10–0.25)	0.20 (0.14–0.38)	0.02
Glycemia (mg/dl)	98 ± 18	103 ± 40	0.11
HbA1c (%)	5.9 ± 0.8	6.1 ± 1.3	0.15
Cardiovascular Score	1.66 ± 1.2	1.70 ± 1.1	0.68
Homocysteine (μmol/l)	15 (12–19)	15 (13–18)	0.40
Vitamin B12 (pg/ml)	308 (252–391)	322 (278–390)	0.18
Folic acid (ng/ml)	5.9 (4.9–7.1)	6.1 (5.1–7.3)	0.46
Comorbidities			
Hypertension (%)	56	58	0.97
Coronary heart disease (%)	13.7	14.2	0.81
Diabetes (%)	17.6	14.5	0.37
Stroke (%)	4.3	4.9	0.78
COPD (%)	6.6	6.2	0.83
MRI parameters			
Cortical lesions (%)	13.5	12.0	0.64
Single lacunar lesion (%)	8	7	0.77
Multiple lacunar lesions (%)	33	37	0.68
Leukoaraiosis (%)	36	40	0.45
Atrophy (%)	56	52	0.43

Continuous variables are expressed as mean ± SEM or median (interquartile range). Categorical variables are expressed as percentage within group

*MMSE* mini mental state examination, *GDS* global deterioration scale, *IADL* instrumental activities of daily living, *BADL* basic activity of daily living, *HDL-C* high density lipoprotein cholesterol, *Hs-CRP* high-sensitivity C-reactive protein, *HbA1c* hemoglobin A1c

In Table 3 are reported the results of univariate Cox regression analysis for the conversion of MCI to dementia, based on the results reported in Table 1. Age (for 1 year increase—hazard ratio: 1.06; 95% confidence interval: 1.02–1.09), MMSE score (for 1 point increase—HR 0.88; 95% CI 0.84–0.93), and type of MCI (amnestic vs non-amnestic—HR 2.38; 95% CI 1.56–3.62) were significantly associated with the progression to dementia. On the contrary, the association between GDS score (three categories; 0–4, 5–10, > 10 points) and progression was not significant.

**Table 3:** Univariate Cox regression models for conversion of MCI to dementia

Variables	*B* (SE)	HR	95% CI	*p*
Age (1 year)	0.057 (0.015)	1.06	1.02–1.09	0.0001
MMSE (1 point)	− 0.12 (0.027)	0.88	0.84–0.93	0.0001
aMCI type	0.87 (0.21)	2.38	1.56–3.62	0.0001
GDS score				
0–4	–	1	–	
5–10	− 0.28 (0.20)	0.75	0.50–1.11	
> 10	− 0.16 (0.30	0.85	0.47–1.54	0.35

*MMSE* mini mental state examination, *GDS* global deterioration scale, *SE* standard error, *HR* hazard ratio, *CI* confidential index

In Table 4 are described the results of Cox multivariate regression model (stepwise forward—Wald) for progression of MCI to dementia. In order of entry into the model, MMSE score (for 1 point increase—HR 0.84; 95% CI 0.79–0.90), amnestic type of MCI (HR 2.35; 95% CI 1.39–3.98), and age (for 1 year increase—HR 1.05; 95% CI 1.01–1.10) were associated with the progression to overt dementia, independent of sex and education; further adjustment for hs.CRP, multiple lacunar lesions and GDS score did not modify the results (data not shown).

**Table 4:** Multivariate Cox regression model (stepwise forward Wald) for conversion of MCI to dementia

Variables	*B* (SE)	HR	95% CI	*R* squared	Total *R* squared	*p*
1. MMSE	− 0.164 (0.034)	0.84	0.79–0.90	24.73	24.73	0.0001
2. aMCI type	0.827 (0.268)	2.35	1.39–3.98	13.20	37.93	0.0001
3. Age	0.057 (0.021)	1.05	1.01–1.10	7.50	45.44	0.0001

Adjusting covariates: age, sex, MCI type, MMSE (continuous variable), education

*MMSE* mini mental state examination, *SE* standard error, *HR* hazard ratio, *CI* confidential index

**Fig. 1:**
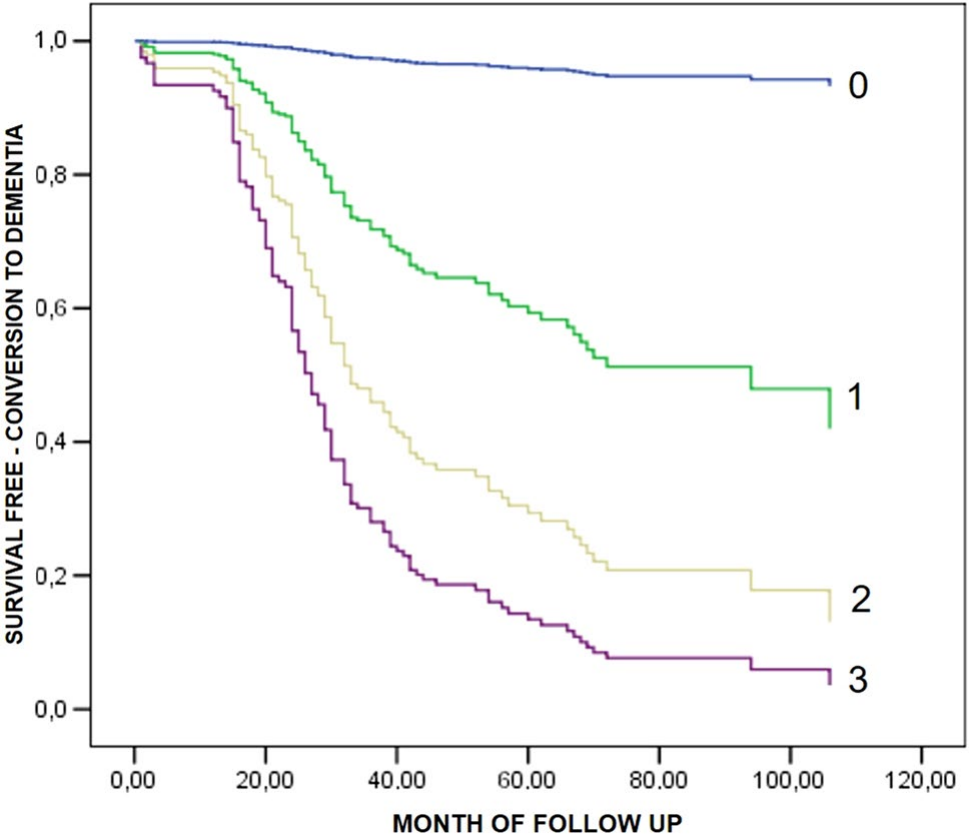
Prognosis of dementia by number of predictors (0–3) including age > 78 years, MMSE < 25/30, and amnestic type of MCI

In Fig. 1 are reported the survival curve obtained from Cox regression model for the progression to dementia in the MCI subjects grouped by the number (0–3) of significant predictors present in each individual: age (≥ or < 78 years, median value), MMSE score (≥ or < 25/30, median value), and aMCI type. Compared to subjects with 0 predictors, a graded and significant increase in the risk of progression to dementia was found in individuals bearing one factor (HR 12; 95% CI 1.7–91), two factors (HR 28; 95% CI 4.0–210) or three factors (HR 45; 95% CI 6.5–328). Interestingly, the conversion rate progressed in a linear way from 6% in subjects with score 0, to 31% in individuals with score = 1, to 53% in subjects with score 2, to 72% in individuals with score 3. The ROC curve analysis for the score showed an area under the curve of 0.72 (95% CI 0.66–0.75) with a standard error 0.030 (*p* 0.0001) (Supplementary Fig. 2).

## Discussion

In this study, we searched for possible predictors of progression to dementia in a large sample of MCI elderly patients, focusing on easily accessible parameters. This is an important topic since the clinical diagnostic category “MCI” is heterogeneous. Indeed, it is known that while a part of individuals will progress to dementia, a large number of MCI will remain “stable” [5], and a part of them will even regress to normal cognitive functions [6].

In our study, about 30% of MCI patients evolved to dementia during the follow-up (median 33 months) with an observed maximum conversion rate between 1 and 3 years from baseline (almost 50% of all cases).

At multivariate analysis, the diagnosis of aMCI was associated with a 135% increase in the probability of progression to dementia compared with naMCI. aMCI displayed a high rate of progression (49%), and predominantly progressed to LOAD (33%) or “mixed” dementia (44%). Conversely, naMCI showed a lower rate of conversion (28%), and mainly evolved to VD (25%) or “mixed dementia” (43%). These findings are in good agreement with previous observations [18, 19]. However, as previously noted by Fischer et al. [20], the subtypes of MCI were not very useful in defining with precision the evolution to different types of dementia; indeed, both aMCI and naMCI progressed to LOAD, while the evolution to VD was not restricted to naMCI.

Age was another independent predictor of MCI conversion, with an increase of the risk of 5% per year at multivariate analysis. In particular, individuals with age ≥ 78 years (median age in our sample) had an 87% risk increase compared with younger subjects. This finding was expected since the incidence of dementia has been associated with age in MCI individuals [18, 21–23].

The degree of the cognitive impairment at baseline was also strongly associated with the progression to dementia; a reduction of 1 point in MMSE score was independently associated with a 16% increase in the risk of progression. Individuals with MMSE score < 25/30 (median value in our sample) displayed an increase of 151% in the risk of developing dementia compared with subjects with MMSE ≥ 25/30. Although our results are in agreement with previous studies [18, 24–26], it has to be noted that a metanalysis by Arevalo-Rodriguez et al. [27] found that the accuracy of baseline MMSE score for conversion to dementia generally was low, with a sensitivity of 23–76% and specificity of 40–94%.

Based on these findings, we created a simple individual prognostic score based on the presence of three parameters including aMCI type, age ≥ 78 years, and MMSE < 25/30. The score stratified our MCI population in four group with increasing risk of future evolution to dementia:

- MCI patients with score 0 (13% of sample) had a very low probability of evolution to dementia, with a very high negative predictive value (0.94). Of consequence, they should be reassured and followed in a less pressing way compared with individuals with higher scores.
- MCI patients with score 1 (28% of sample) had a low probability of evolution to dementia, with a high negative predictive value (0.69).
- MCI patients with score 2 (42% of sample) had an intermediate probability of progression, with a high positive predictive value (0.53).
- MCI patients with score 3 (17% of sample) had a high probability of progression, with a high positive predictive value (0.72). These subjects should be deeply investigated to exclude a possible underlying diagnosis of initial dementia. Moreover, these patients might gain much more benefit from specific therapies, although at present no drugs are specifically approved for MCI treatment.

We also calculated a cardiovascular risk score based on well-known risk factors and vascular comorbidities (CHD and stroke). Although the cardiovascular risk has been associated with both MCI [28], and progression from MCI to dementia [29] in our sample the cardiovascular risk score was not predictive of the progression to dementia.

Finally, we must acknowledge an important limitation of the study. In evaluating the risk of progression to dementia we did not include some comorbidities among possible confounders, such as heart failure and atrial fibrillation. Indeed, a continuum among hypertension, coronary artery disease, atrial fibrillation, and chronic heart failure with the development of cognitive impairment and progression to dementia has been hypothesized [30]. Moreover, it has been demonstrated not only that atrial fibrillation predicts evolution to dementia in elderly subjects with MCI, but also that ventricular rate response seems to play a key role in dementia incidence among patients with atrial fibrillation [31].

We would also underline some strength of the study, in particular the large size of the MCI sample (over 450 individuals) and the length the follow-up (median length 33 months).

## Conclusion

In conclusion, we have described a simple score, based on previously recognized predictors such as age, MMSE score, and type of MCI, which may be useful for an initial stratification of the risk of progression to dementia in patients affected by MCI. The score might help the clinician to evaluate the need for more expansive/invasive examinations and for a closer follow-up in MCI patients.

## Electronic supplementary material

Below is the link to the electronic supplementary material.

Supplementary Fig. 1: Rate of progression to dementia of amnesitic MCI (aMCI) non-amnestic MCI (naMCI), multidomain naMCI, single-domain naMCI (12%) (DOCX 92 kb)

Supplementary Fig. 2: Receiver operating characteristic (ROC) curve analysis for prognostic score showing an area under the curve of 0.72 (95%CI 0.66–0.75) with a standard error 0.030 (p: 0.0001) (DOCX 33 kb)
