# Supplementary figures and images for: Clinical and demographic parameters predict the progression from mild cognitive impairment to dementia in elderly patients
Source: Aging Clin Exp Res. 2020 Sep 12;33(7):1895–902. doi: 10.1007/s40520-020-01697-8 (PMC8249246; doi:10.1007/s40520-020-01697-8)

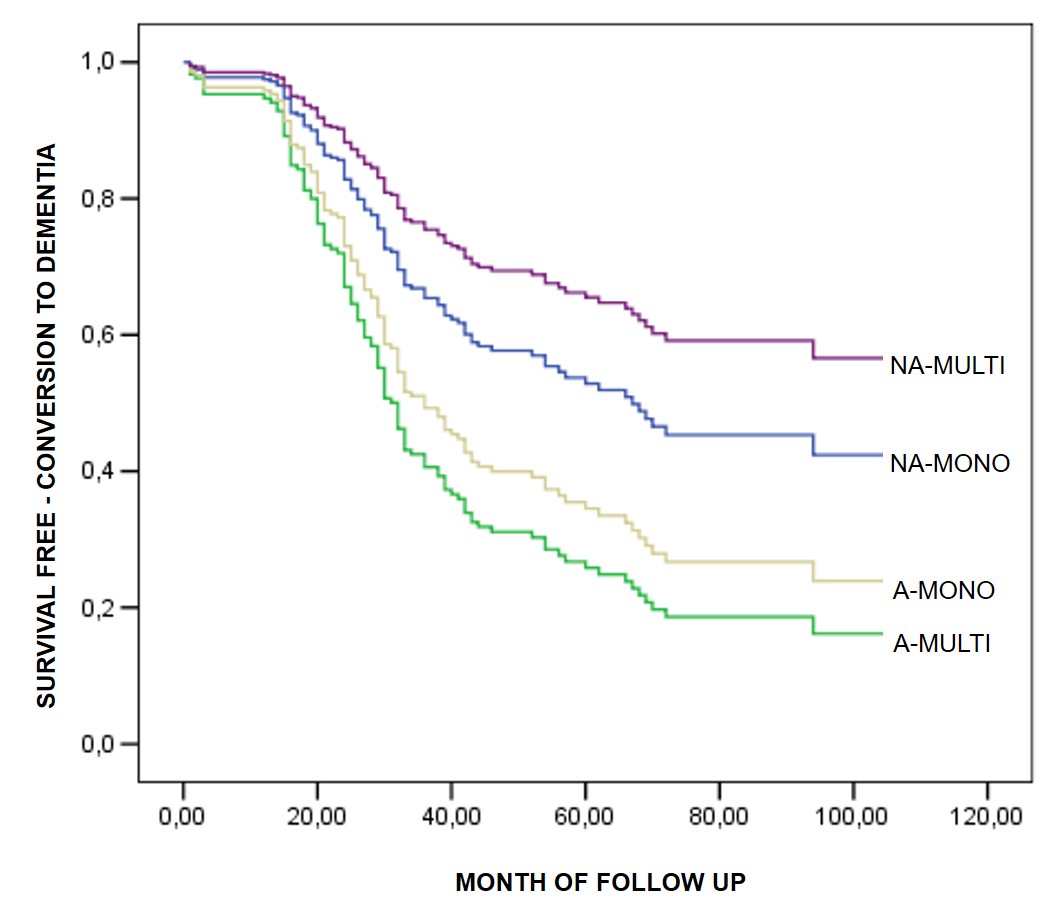

Supplement: Supplementary file 1 — Supplementary Fig. 1: Rate of progression to dementia of amnesitic MCI (aMCI) non-amnestic MCI (naMCI), multidomain naMCI, single-domain naMCI (12%) (DOCX 92 kb) [file 40520_2020_1697_MOESM1_ESM.docx]

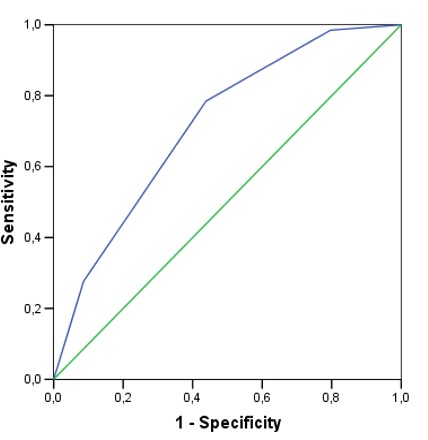

Supplement: Supplementary file 2 — Supplementary Fig. 2: Receiver operating characteristic (ROC) curve analysis for prognostic score showing an area under the curve of 0.72 (95%CI 0.66–0.75) with a standard error 0.030 (p: 0.0001) (DOCX 33 kb) [file 40520_2020_1697_MOESM2_ESM.docx]
